# Physiotherapy informed by Acceptance and Commitment Therapy (PACT): protocol for a randomised controlled trial of PACT versus usual physiotherapy care for adults with chronic low back pain

**DOI:** 10.1136/bmjopen-2016-011548

**Published:** 2016-06-07

**Authors:** Emma Godfrey, Melissa Galea Holmes, Vari Wileman, Lance McCracken, Sam Norton, Rona Moss-Morris, John Pallet, Duncan Sanders, Massimo Barcellona, Duncan Critchley

**Affiliations:** 1 Department of Psychology, Institute of Psychiatry, Psychology and Neuroscience, King's College London, London, UK; 2 Faculty of Life Sciences and Medicine, Division of Health and Social Care Research, Department of Physiotherapy, King's College London, London, UK; 3 Pain Management Research Institute, Sydney Medical School—Northern, Royal North Shore Hospital, Sydney, New South Wales, Australia; 4 King's College Hospital NHS Foundation Trust, London, UK; 5 Faculty of Life Sciences and Medicine, Division of Health and Social Care Research, Department of Physiotherapy, King's College London, London, UK

**Keywords:** PAIN MANAGEMENT

## Abstract

**Introduction:**

Chronic low back pain (CLBP) is a common condition and source of significant suffering, disability and healthcare costs. Current physiotherapy treatment is moderately effective. Combining theory-based psychological methods with physiotherapy could improve outcomes for people with CLBP. The primary aim of this randomised controlled trial (RCT) is to evaluate the efficacy of Physiotherapy informed by Acceptance and Commitment Therapy (PACT) on functioning in patients with CLBP.

**Methods and analysis:**

The PACT trial is a two-armed, parallel-group, multicentre RCT to assess the efficacy of PACT in comparison with usual physiotherapy care (UC). 240 patients referred to physiotherapy with CLBP will be recruited from three National Health Service (NHS) hospitals trusts. Inclusion criteria are: age ≥18 years, CLBP ≥12-week duration, scoring ≥3 points on the Roland-Morris Disability Questionnaire (RMDQ) and adequate understanding of spoken and written English to participate. Patients will be randomised to PACT or UC (120 per arm stratified by centre) by an independent randomisation service and followed up at 3 and 12 months post randomisation. The sample size of 240 will provide adequate power to detect a standardised mean difference of 0.40 in the primary outcome (RMDQ; 5% significance, 80% power) assuming attrition of 20%. Analysis will be by intention to treat conducted by the trial statistician, blind to treatment group, following a prespecified analysis plan. Estimates of treatment effect at the follow-up assessments will use an intention-to-treat framework, implemented using a linear mixed-effects model.

**Ethics and dissemination:**

This trial has full ethical approval (14/SC/0277). It will be disseminated via peer-reviewed publications and conference presentations. The results will enable clinicians, patients and health service managers to make informed decisions regarding the efficacy of PACT for patients with CLBP.

**Trial registration number:**

ISRCTN95392287; Pre-results.

**Strengths and limitations of this study**

- The Physiotherapy informed by Acceptance and Commitment Therapy (PACT) trial will be the first randomised controlled trial to test the efficacy of a physiotherapist-led ACT-informed intervention for chronic low back pain (CLBP) against standard physiotherapy.
- The PACT trial will assess the feasibility of training physiotherapists to deliver a novel psychologically informed physiotherapy intervention.
- Theory-based processes of change consistent with the psychological flexibility model will be evaluated, providing evidence for the mechanisms underpinning observed outcomes.
- Restriction to participants referred to physiotherapy services and speaking English may limit generalisability of findings.
- Patients who have had prior treatment from multidisciplinary or cognitive–behavioural therapy (CBT) pain management at any time and other physiotherapy treatment in the previous 6 months will be excluded due to possible contamination effects.

## Introduction

Low back pain has a lifetime prevalence ranging from 60% to 70% in industrialised countries, causes more years of disability than any other health condition and is the second most frequent reason for absence from work.^1^ ^2^ Chronic low back pain (CLBP) is pain that has lasted for more than 12 weeks. It causes considerable suffering to the individual and is a major financial burden on the National Health Service (NHS) and wider society. UK healthcare costs are £1.6 billion annually,^3^ and CLBP is responsible for 80% of this cost.^4^

Physiotherapy is a common treatment for CLBP, with 1.26 million patients referred to NHS physiotherapists at a cost of £150 million per annum.^5^ Several forms of physiotherapy are recommended for CLBP, including exercises, manual therapy and back classes.^6^ The type of physiotherapy delivered varies considerably in duration and content, and there is little consensus about the most appropriate and cost-effective treatment.^7^ ^8^ Many trials show no clear superiority for any treatment, with the majority leading to no more than modest improvement in pain and disability outcomes.^9^ As a result, patients are often overtreated, placing high demands on physiotherapy services and delaying active self-management. This highlights the need to develop and test novel treatments for patients with CLBP.^10^

CLBP is best suited to a biopsychosocial model of care^11^ and a cognitive behavioural approach to treatment.^12^ Cognitive–behavioural therapy (CBT) has a good evidence base for the treatment of chronic pain.^13–15^ A Cochrane review concluded that further general randomised controlled trials (RCTs) of CBT for chronic pain were not required.^16^ Instead, studies identifying the specific components of CBT and attempting to understand which underlying processes were successful were recommended. The Chartered Society of Physiotherapy recognises that CBT can fall within a physiotherapist's scope of practice.^17^ However, CBT-based treatments delivered by physiotherapists have only produced moderate improvements in CLBP-related disability,^18^ ^19^ and many physiotherapists do not feel adequately trained to use psychological techniques effectively.^20^ There is potential for enhancing effectiveness through greater focus on competency, but it remains unclear how to best implement cognitive and behavioural approaches during physiotherapy interventions.

One promising theory-based approach to chronic pain is a form of CBT called Acceptance and Commitment Therapy (ACT).^21^ ^22^ ACT has been shown to have positive effects in chronic pain,^23^ ^24^ and meta-analyses of ACT for chronic pain showed improvements in depression, anxiety, pain intensity, physical functioning and quality of life (QoL).^25^ ^26^ ACT aims to increase psychological flexibility and focuses on improving function rather than reducing pain. It has good maintenance of treatment effects up to 3 years post treatment,^27^ important in a chronic relapsing and remitting condition such as CLBP. In all published studies to date, ACT has been delivered by psychologists or within multidisciplinary teams; however, psychology is a limited resource and most patients with CLBP are seen by physiotherapists. A recent trial of ACT for CLBP delivered by psychologists found that patients referred for physiotherapy were somewhat resistant to seeing a psychologist and consequently has recommended combining ACT with physiotherapy.^28^ A qualitative study investigated potential barriers and facilitators to embedding ACT within a physiotherapist-led pain rehabilitation programme. Findings suggested this presented challenges and opportunities but was a positive experience overall if extra support was provided.^29^

We have developed a brief physiotherapist-delivered treatment, guided by principles of ACT, Physiotherapy informed by Acceptance and Commitment Therapy (PACT), consisting of two face-to-face sessions plus a follow-up telephone call. A small proof-of-concept feasibility study demonstrated the acceptability of the intervention for patients, and recruitment to a larger trial was achievable.^30^ This protocol describes a phase II efficacy RCT using a two-armed, parallel-group design to assess the efficacy of PACT for improving function at 3 months in individuals with CLBP, in comparison with usual physiotherapy treatment. Across three NHS trusts (including six hospital centres), 240 people with CLBP will be individually randomised to PACT or usual physiotherapy care (UC). We hypothesise that the group receiving PACT will have improved self-reported functioning at the primary end point of 3-month follow-up compared with UC. The PACT trial is funded by the National Institute for Health Research (NIHR) Research for Patient Benefit programme, reference number PB-PG-1112-29055.

## Methods and analysis

### Main research question

What is the efficacy of PACT for improving functioning in patients with CLBP?

### Research objectives

#### Primary objectives

1. The primary objective of this study is to evaluate the efficacy of PACT on the primary end point of functioning at 3-month follow-up.

#### Secondary objectives

1. To assess whether PACT has a positive impact on secondary outcomes: QoL and function in various domains, process variables such as acceptance and committed action, mood, self-efficacy and pain compared with UC at 3-month and 12-month follow-up.
2. To investigate optimal ways of training physiotherapists to work in extended roles and develop a PACT training package for use in a definitive multicentre trial.
3. To pilot methods and instruments needed to estimate cost-effectiveness in a future phase III trial from a health service and societal perspective.
4. To assess the acceptability of the intervention and training for patients and clinicians via nested qualitative studies.
5. To investigate hypothesised processes of clinical improvement following PACT, including predictors and moderators of outcome, and treatment fidelity.

### Design

A phase II, assessor-blind, multicentre, two-armed, parallel-group RCT.

### Method

A total of 240 patients with CLBP will be individually randomised to PACT or UC.

### Setting

Participants will be recruited from secondary care physiotherapy clinics in two NHS Foundation Hospital trusts in London (Guy's and St Thomas' and King’s College Hospital) and one in the South East of England (Ashford and St Peter's), UK. Treatment will take place in the physiotherapy clinics based at the participating hospitals.

### Eligibility

*Inclusion criteria*: Adults (aged 18 years and over) with non-specific CLBP (confirmed by a clinical physiotherapist) of >12 weeks duration, with or without associated leg pain and reporting a score of 3 points or more on the Roland-Morris Disability Questionnaire (RMDQ). Patients need to be able and willing to provide informed consent and attend treatment at hospital. Potential participants require a good understanding of spoken and written English to complete trial data collection and participate in the PACT programme.

*Exclusion criteria*: Prior treatment from multidisciplinary CBT pain management at any time and other physiotherapy treatment in the previous 6 months or injection therapy within 3 months. Specific medically diagnosed lumbar spine pathology (eg, inflammatory arthritis, fracture or cancer). Patients with deteriorating neurological signs (stable neurological signs and pain of apparently neuropathic origin are not exclusion criteria) and those with previous experience of or awaiting spinal surgery. Patients with current psychiatric illness (eg, severe depression, personality disorder or post-traumatic stress disorder) and/or current drug or alcohol misuse likely to interfere with treatment.

*Withdrawal criteria*: Participants will be withdrawn from the trial if there are any concerns regarding informed consent. Participants can also withdraw if they choose to without giving a reason. If patients withdraw consent for research follow-up during the trial, reasons for dropout will be recorded where possible.

### Planned interventions

#### Physiotherapy informed by Acceptance and Commitment Therapy

PACT is a brief physiotherapy intervention guided by principles of ACT designed to promote self-management, consisting of two 60 min face-to-face sessions 2 weeks apart, plus one booster telephone call (lasting 20 min), 1 month after the last treatment session. PACT alters the content of physiotherapy treatment and reconfigures it so that it is delivered in fewer but longer sessions, although the total contact time is similar to the average amount of time patients with CLBP receive as part of usual physiotherapy treatment as reported in two UK RCTs for CLBP, where usual physiotherapy was used as the control arm.^31^ ^32^ Two 60-min sessions are designed to allow adequate time to do an initial physical assessment and provide feedback, create value-based goals, provide individualised physical exercises and teach simple psychological skills to promote psychological flexibility, and to address facilitators and barriers to self-management. The booster phone call promotes self-management by giving patients a chance to feedback progress and gain support with any ongoing issues they may have. PACT thus aims to directly reduce avoidance and promote openness, to build present-focused awareness, and coordinate greater engagement in goal-oriented and value-based activity (see box 1 below). The face-to-face intervention will be supported by a patient manual individualised to patient needs. Patients randomised to PACT will be given their patient manual during their first session.

Box 1 **Summary of the content of Physiotherapy informed by Acceptance and Commitment Therapy (PACT) sessions**

*PACT Session 1: 60 min face to face*

- Set the agenda: outline structure, schedule and delivery of treatment.
- Assessment, feedback and rationale: conduct brief physical assessment and discuss results. Empathise with and normalise current feelings and provide guidance that no serious medical problems have been uncovered and it is safe to resume normal activities.
- Shifting focus from pain to function: discuss previous attempts to reduce pain, which are not usually very successful in relation to daily functioning. Build open engagement rather than struggling with pain. Present the goal of PACT, to help people function better, especially in the areas that are important to them. Use metaphors to help make this shift.
- Value-based goal setting: introduce patient manual. Engage patient in identifying core values and setting related goals. Break goals down into small steps that are positive, practical and achievable, and record these in the manual.
- Skills training to address barriers to goal attainment: implement strategies to promote openness, awareness and engagement, for example, mindfulness exercises, action plans and making a public commitment to goals, to help anticipate and overcome perceived barriers to change.

*PACT Session 2: 60 min face to face*

- Review successes and challenges: positively reinforce progress towards goals, discuss how this was achieved and highlight benefits. Review, normalise and empathise with challenges and encourage continued use of the patient manual.
- Goal adjustment/development: check the salience of goals and make adjustments if required. Re-establish commitment using motivational interviewing techniques if necessary. Use exercises and metaphors to normalise setbacks, keep moving in small steps toward goals and troubleshoot or prevent the effects of barriers.
- Generalisation to new areas: rehearse new skills, such as mindfulness and shifting focus and explore how these can be extended to other areas of life. Encourage the development of insights and the capacity to self-initiate change.
- Integration of self-management approach: review key skills and identify a support network. Discuss maintenance tools and again normalise setbacks.

*PACT Booster Call: 20-min phone call*

- Review progress: appreciate successes to date and discuss any remaining barriers.
- Assessment of skills integration into everyday life: review key skill sets so that they organise the participant's learning in the areas of openness, awareness and engagement.
- Support generalisation: build on patterns of initial goal achievement and broaden the scope of applications to other areas.
- Reinforce continued self-management: emphasise to the patient that they will face times in the future when they experience pain or other difficulties and they have resources to deal with this, such as the patient manual and new skills. Positive closure of the therapeutic partnership to help reinforce their capacity to persist with the tools they have to manage their back pain without needing more healthcare.

### Training and supervision

PACT will be delivered by 8 Band 6 or 7 trial physiotherapists (2 per centre). Physiotherapists will be identified by their managers and invited to volunteer to take part in the study. The physiotherapist will then be sent information about the PACT study and be invited to meet the study team to discuss their participation. Training will be provided by LM, a clinical psychologist and expert in ACT, with the assistance of EG, a health psychologist, and DC, a physiotherapist, before the start of recruitment. Group face-to-face training including experiential exercises and role play will last 2 days and will be supported by a manual. The manual consists of an introduction to ACT and promoting behaviour change; information about the trial; strategies, metaphors and skills to enable PACT delivery; detailed session plans (see box 1); explanation of competency and fidelity, including the use of supervision and a reflexive diary. Obstacles to therapist and patient engagement and progress will be discussed, as well as strategies for dealing with these eventualities. The trial protocol will be reviewed, including recording the timing and length of sessions, any deviations from protocol including missed sessions or dropout, and confidential storage of audio recordings. A training package will be further developed through feedback forms filled in at the end of the group face-to-face training and via interviews with all trained physiotherapists as part of this study, to enable its use in a larger phase III trial if PACT is successful. Each physiotherapist will practise delivering PACT and receive at least two sessions of individual supervision to ensure adequate competency to commence treatment. As PACT is a novel treatment, we will assess competency qualitatively through the training and initial supervision process, which will include listening to audiotaped sessions and observing role play. Physiotherapists will also be asked to report back on experiences with practice patients before they start the trial. If a physiotherapist is not deemed competent to begin delivery after two individual sessions of supervision, they will be offered more sessions until a satisfactory level of competency is observed. We will continue to assess competency throughout the trial on a monthly basis. It is assumed competency will improve during the course of delivery as skills are enhanced through practice and supervision. Trial physiotherapists will attend monthly supervision meetings with supervisors (LM, EG and DC), to maintain skills and receive support. Regular supervision will ensure that the physiotherapists adhere to the trial protocol and that the quality of the intervention is maintained. Fidelity to the treatment protocol will also be enhanced by the use of session checklists and ratings of audio tapes from the trial with feedback provided to clinicians.

### Treatment fidelity

All PACT sessions will be audio-recorded for the purpose of assessing treatment fidelity. These will be used for supervision during the study and to check fidelity throughout the trial. Supervisors will listen to one tape per physiotherapist per month. Once the trial has ended, a subset of the audio recordings will be analysed for overall fidelity. These fidelity checks will be undertaken via assessment of a sample of audio recordings of PACT sessions, across sites and physiotherapists, undertaken by two independent researchers. A modified fidelity measure will be developed based on the Plumb and Vilardaga^33^ paper and the ACT for Chronic Pain Adherence Rating Scale used in the Optimised Behavioural Intervention trial.^28^ At least two sessions from every physiotherapist will be rated in terms of adherence to the manual and checklist. The therapeutic alliance between physiotherapists and participants will also be rated using a therapy process scale^34^ employed in previous RCTs of treatments for chronic fatigue and a weight loss intervention in primary care.

#### Usual care

Participants randomised to UC will receive any treatment considered suitable by their treating physiotherapist. Treatment may include any type of individual physiotherapy and/or back classes, for example, exercises, manual therapy, hydrotherapy and back schools. Consistent with the Consolidated Standards of Reporting Trials (CONSORT) guidelines for complex interventions,^35^ we will collect data on volume (duration and frequency of sessions) and components (eg, one-to-one treatment vs group exercise class) of treatment received by participants in the UC arm, and we plan to report and publish these essential details of the control condition with trial results.

Separate groups of clinicians will deliver PACT and UC to avoid contamination. We will explicitly inform PACT physiotherapists about the risks and consequences of contamination during training and supervision and will ask that they do not share material or ideas with their colleagues during the treatment delivery period. In addition, we will ensure that all PACT sessions are conducted in private rooms to eliminate the possibility of UC physiotherapists overhearing what is being provided in the novel treatment arm.

### Participant identification and recruitment

**Figure 1:**
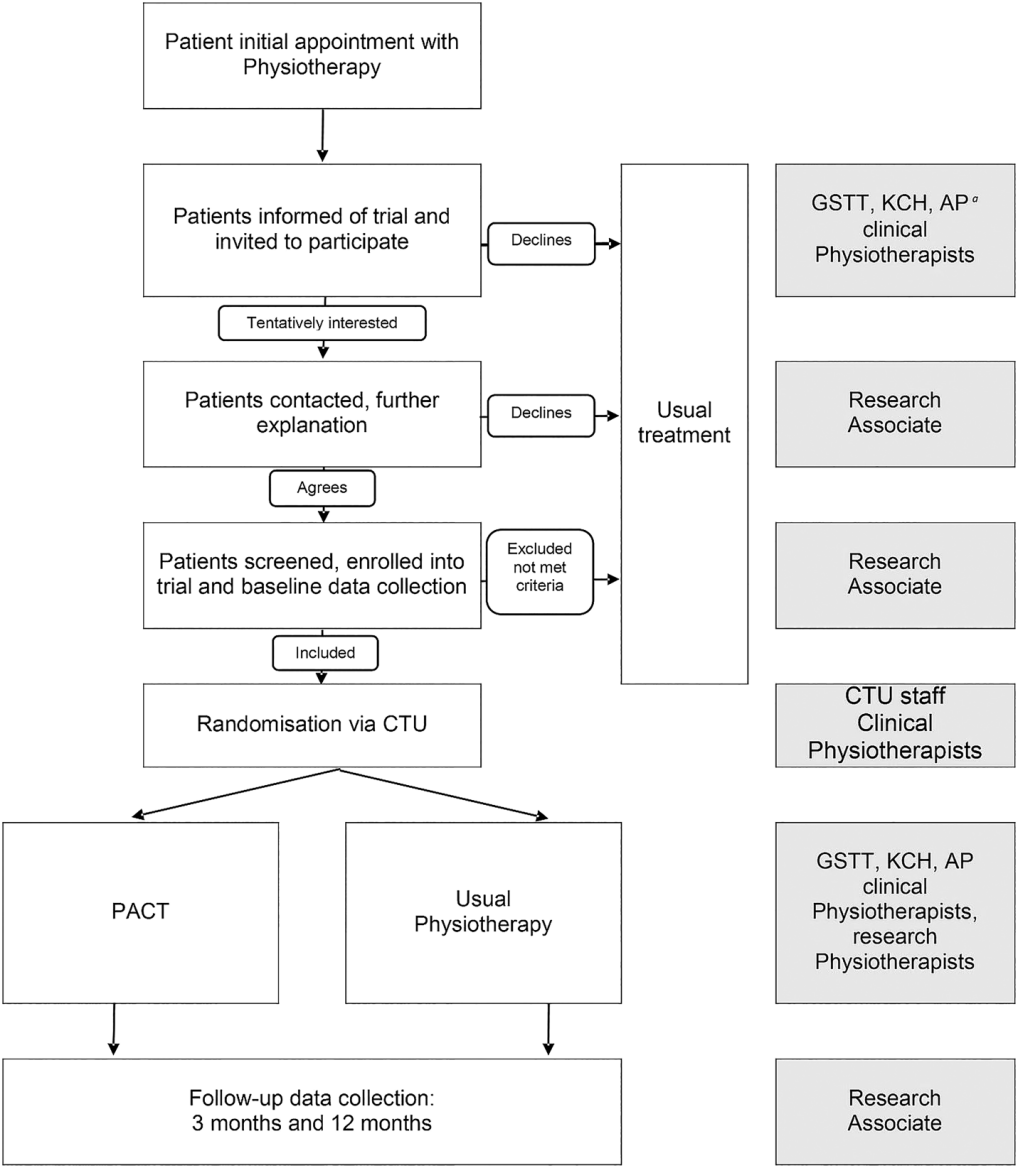
PACT trial process flow chart. CTU, King's Clinical Trials Unit; PACT, Physiotherapy informed by Acceptance and Commitment Therapy. ^*a*^Guy's and St Thomas' Hospitals, King's College Hospital and Ashford and St Peters Hospitals.

In total, 240 patients will be recruited, 120 per treatment arm, over an 18-month period. Patients will be recruited from six secondary care physiotherapy clinics in London and the South East of England. Posters advertising the study will be placed in relevant physiotherapy clinics in order to inform patients and clinicians about the study. Potential participants referred to outpatient physiotherapy by their general practitioner (GP) or consultant will be identified by clinical physiotherapists from each hospital centre at their initial triage sessions, provided with written and verbal information about the PACT trial, and invited to participate. Participants who consent to be contacted will be referred to the research associates (RAs) for full eligibility screening, conducted by telephone. All patients who undergo screening will be recorded anonymously on a screening database associated with the study by the RAs. If the patient is suitable, the RA will then invite them to complete consent forms and baseline measures either at home online, via postal questionnaires or in person at the clinic (table 1). GPs will be notified in writing of their patient's participation. Patients will be informed that they can withdraw from the study at any time without giving a reason and that this will not affect the treatment they receive in any way. All participant responses will be anonymous and confidential, and participants will not be identified in any way by their responses (figure 1).

**Table 1:** Screening and data collection across the trial: summary of the key trial processes from a potential participant agreeing to be contacted to the data collection time points

Process	Completed by	Format of administration	Pre-consent	Baseline	3-Month follow-up	12-Month follow-up	Ongoing during treatment period	Reference
Identification*	PT	PP	●					
Screening	RA	Telephone	●					
Consent	P	PP/DB		●				
Randomisation	CTU	CTU Database		●				
Sociodemographics	P	PP/DB		●				
EuroQol-5D-5L	P	PP/DB		●	●	●		36
Medical Outcomes Survey Short Form-12 (V.2)	P	PP/DB		●	●	●		37
Roland-Morris Disability Questionnaire	P	PP/DB		●	●	●		38
Chronic Pain Acceptance Questionnaire-8	P	PP/DB		●	●	●		39
Committed Action Questionnaire-8	P	PP/DB		●	●	●		40
Numeric Analogue Scale	P	PP/DB		●	●	●		–
Patient-Specific Function Scale	P	PP/DB		●	●	●		41
Work and Social Adjustment Scale	P	PP/DB		●	●	●		42
Pain Self-Efficacy Questionnaire	P	PP/DB		●	●	●		43
Generalised Anxiety Disorder-7	P	PP/DB		●	●	●		45
Life Satisfaction Scale	P	PP/DB		●	●	●		–
Patient Health Questionnaire-9	P	PP/DB		●	●	●		46
Global improvement	P	PP/DB			●	●		47
Satisfaction with outcome	P	PP/DB			●	●		47
Treatment credibility	P	PP/DB			●	●		48
Health-related resource use	P	PP/DB		●	●	●		49
Self-reported adverse event	P	PP/DB			●	●		–
Clinician-reported adverse event	PT	PP/DB					●	–
Treatment attendance	PT	DB					●	–

*Includes permission to contact and provision of patient information letter.

CTU, King's Clinical Trials Unit; DB, online database; P, participant; PP, paper and pencil; PT, physiotherapist; RA, research associate.

### Study procedures

Information on study procedures is summarised in the CONSORT diagram (figure 1) and table 1 (screening and data collection).

### Randomisation

Randomisation will be provided by an independent randomisation service at the UK Clinical Research Collaboration (UKCRC)-registered King's Clinical Trials Unit (CTU). Randomisation will be at the level of the individual, using block randomisation with randomly varying block sizes, stratified by centre and implemented via the King's CTU online system, with emails generated automatically and sent to relevant physiotherapy staff at study sites.

### Blinding

It is not possible to blind patients or treating physiotherapists to the treatment allocation; however, no hypotheses have been proposed to participants as to the superiority of PACT over UC and all participants will receive physiotherapy treatment. The patient information sheet will deliberately maintain a position of equipoise by stating, ‘Each group will get a treatment that we think might be helpful, but we don't know whether one treatment is going to be more helpful than another’.

The RAs screening patients and collecting data and the statistician analysing the data and assessing outcome will be blinded to treatment allocation. All outcomes are patient reported and collected via the internet following automated email reminders, reducing the risk of unblinding the assessors. Locked codes will be used for treatment allocation, and the trial statistician will analyse the data blind.

### Data collection

Participant screening data will be collected by telephone and entered into the database by the RAs. Baseline, 3-month and 12-month follow-up data will be collected through self-report questionnaires. The RAs, blind to treatment allocation, will administer questionnaires and conduct data entry. Treatment allocation will not be included on the questionnaires. Research data will be entered onto the MedSciNet database system, a regulatory compliant database that has been enabled for online collection of patient-reported outcome measures (PROMS). Participants will be given a unique username and password to log into the online database and complete consent and measures at each time point. Their data will be identified by a unique identification number and will be kept separate from any personal identifying data to maintain confidentiality. Baseline and outcome data will be patient self-completed at home (either online or via postal questionnaires), thus avoiding any influence of the study team on the responses and reducing bias. PACT physiotherapists will have access to a unique database on the MedSciNet system to record details of who provided PACT and UC sessions, the number of sessions attended and any dropouts, as well as the number and type of UC treatment sessions attended.

### Outcome measures

*Time points:* Assessments will be completed at baseline (immediately pre-randomisation), and 3 months and 12 months post randomisation by all participants. All time points will be taken into account during analysis, but the primary efficacy end point is 3-month follow-up. In order to justify treatment costs, clinically significant treatment effects need to be maintained over time, and this is particularly important in a chronic relapsing and remitting condition such as CLBP, so maintenance of any treatment effects will be assessed at 12 months. The RAs will be employed to coordinate the trial and collect baseline and follow-up data. All participants will be sent (emailed and posted) follow-up questionnaires by the RAs at 3 and 12 months. Participants not returning questionnaires within 1 week will receive a reminder email, telephone call and text in 3-day intervals. One week after that, if no data have been returned, the RAs will ring the participant to ask if they can collect primary outcome data over the telephone.

### Baseline measures

Participants will complete a baseline assessment questionnaire which includes the validated scales detailed below, plus demographic data to establish sociodemographic characteristics of participants as follows: age, sex, height, weight, self-reported ethnicity, education level, employment and benefit status, diagnosis and history of any medical condition if available.

#### Primary outcome: the RMDQ

Patient-reported disability is recommended as a core outcome measure in low back pain^14^ and chronic pain trials.^50^ The Roland-Morris Disability Questionnaire (RMDQ)^38^ is a 24-item questionnaire assessing self-reported functioning and disability due to CLBP, ranging from 0 (no disability) to 24 (maximum disability). The RMDQ is a widely used and valid measure with good test–retest reliability. A 2–3 point change from baseline is considered clinically important.^51^

#### Secondary outcome measures

Secondary outcomes have been selected to determine the wider effects of PACT and to assess therapeutic processes and mechanisms of action. The outcomes include all core domains recommended in chronic pain research:^50^ pain, function, mood, QoL and satisfaction with treatment. A Global Improvement scale^47^ and Treatment Credibility^48^ Questionnaire will be completed at follow-up.

##### QoL: Work and Social Adjustment Scale (WSAS) and EQ-5D-5L

The Work and Social Adjustment Scale (WSAS) measures the effect of CLBP on participants' ability to work and participate in social and private leisure activities.^42^ The WSAS has five items scored from 0 (not affected) to 8 (severely affected), with a total possible score of 40. The EQ-5D-5L is the most frequently used tool for generating quality-adjusted life years (QALYs), which are favoured by the National Institute for Health and Care Excellence (NICE).^52^

##### Pain: pain Numeric Analogue Scale (NAS)

A single pain item rated using a numerical analogue scale anchored at 0 with ‘no pain’ and 10 with ‘worst possible pain’ will reflect the participants' subjective experience of pain.

##### Function: Patient-Specific Functional Scale (PSFS)

The PSFS is a self-reported measure used to identify and investigate functional status tailored to the patient.^41^ The patient identifies three activities limited by their CLBP, rating them on a scale of 0 (unable to perform activity) to 10 (able to perform activity at the same level as before injury/problem). The scores across the three items are summed to give a total possible score of 30.

##### Mood: Generalised Anxiety Disorder-7 (GAD-7) and Patient Health Questionnaire-9 (PHQ-9)

The GAD-7^45^ has seven items that assess anxiety in the last 2 weeks. Scores range from 0 to 21, with a total score of ≥8 indicating probable generalised anxiety disorder. The PHQ-9^46^ is a brief nine-item questionnaire that identifies and quantifies depressive symptoms; scores range from 0 to 27, with a total score of ≥10 indicating probable depressive disorder. Both questionnaires are well validated, commonly used self-report instruments for detecting distress, depression and anxiety in patients with medical illnesses.

#### Process variables

##### The Chronic Pain Acceptance Questionnaire-8 (CPAQ-8) and Committed Action Questionnaire-8 (CAQ-8)

Acceptance of pain and persistent but flexible behaviour towards achieving a goal form part of the ACT model and are therefore putative mediators of the efficacy mechanism in PACT treatment. The CPAQ-8^39^ is a shortened version of the original 20-item Chronic Pain Acceptance Questionnaire, which assesses the capacity to engage in activities without struggling with the pain. Each item is scored from 0 (never true) to 6 (always true), with a total possible score of 48. The CAQ-8^40^ is a shortened version composed of eight questions from the original 18-item Committed Action Questionnaire aimed to measure committed action in terms of commitment to valued goals. The items are rated from 0 (never true) to 6 (always true), with a total possible score of 48.

##### Pain Self-Efficacy Questionnaire (PSEQ)

The PSEQ^43^ assesses confidence in undertaking normal activities despite pain, which is an important variable to measure in interventions designed to enhance self-management. The questionnaire consists of 10 items rated on a 7-point scale anchored at 0 with ‘not at all confident’ and 6 with ‘completely confident’. Items are summed to generate a total possible score of 60.

#### Satisfaction: satisfaction with life, global improvement, treatment credibility

Satisfaction with treatment will be assessed by patients rating their overall improvement in terms of Patient Global Impression of Change (PGIC), their satisfaction with outcome and how credible they found their treatment.^47^ This has five items scored on 11-point scales ranging from 0 (not at all) to 10 (completely). Life satisfaction will be assessed by a single item: ‘All things considered, how satisfied are you with your life as a whole nowadays?’ Responses are on a scale from 0 (extremely dissatisfied) to 10 (extremely satisfied).

#### Health economics: EQ-5D-5L and SF-6D

This study is an important opportunity to pilot methods for estimating the economic impact of interventions on CLBP needed to design cost-effectiveness analyses (CEAs) in future definitive trials. Previously reported CEAs in the UK have relied on utility values derived from two different instruments—the EQ-5D-5L^36^ ^52^ ^53^ and the SF-6D.^18^ The EQ-5D-5L is the most commonly used tool for generating QALYs; however, the SF-6D, which is derived from scores obtained on the Medical Outcomes Survey Short Form-12 (v2), may be more sensitive to change in CLBP. The economic burden of CLBP is considerable from an NHS and patient perspective. A resource use questionnaire which identifies key cost drivers (NHS and non-NHS) will be developed based on previous studies. This will then be piloted to ensure completion rates, avoid redundant questions and to identify any additional resource use items sensitive to change in a CLBP population.^54^ This pilot study will compare the validity and sensitivity of the EQ-5D-5L (the most recent version of the EQ-5D) and the SF-6D for use in economic evaluations of cognitively enhanced physiotherapy for CLBP.

#### Adherence to PACT treatment

Patients' adherence to PACT treatment will be recorded by trial physiotherapists on a database. Attending both face-to-face sessions will be considered adherence to PACT treatment. In the UC arm, the type of treatment and attendance at physiotherapy sessions will be recorded by the trial physiotherapists on the database. Any modifications or departures from randomised treatments, withdrawal of participants from trial treatment or research follow-up will be recorded and reported as such.

#### Qualitative component

##### Patient interviews

A nested qualitative study will explore patients' experiences of PACT treatment. The aim of these methods will be to assess patients' views of the acceptability of PACT, to provide insight into the quantitative results and to explore processes of change. Semistructured face-to-face interviews will be conducted with up to 25 participants (sampled purposively to encompass a mix of gender, age, recruitment site and baseline RMDQ scores) following their 3-month follow-up assessment (RCT outcome primary end point). Interviews will be transcribed verbatim and analysed using thematic analysis^55^ to generate the key themes. Analysis will commence after the first interview in an iterative process, allowing early insights to be explored more fully in later interviews and topic guides to be amended as necessary. A reflexive diary will be kept during the recruitment, interview and data analysis process to ensure transparency of the analysis process. Respondent validity and independent coding by another researcher will be conducted to check the validity of emergent themes.

##### Physiotherapist interviews

Additional, nested, longitudinal, qualitative methods will explore the feasibility and acceptability of the PACT training programme for physiotherapists. All physiotherapists trained in PACT will be invited to attend individual face-to-face semistructured interviews by independent researchers. Later, the eight physiotherapists providing PACT treatment will be interviewed on two more occasions, 6 months after training and at the end of treatment delivery, to assess their perceptions of delivering this novel physiotherapy service treatment. All qualitative interviews will provide insight into the acceptability and feasibility of PACT, development of competency and any contextual factors linked to delivery to inform in any future research in this area.

Interviews will be conducted by independent researchers, transcribed verbatim and analysed using framework analysis to generate the key themes.^56^ Analysis will commence after the first interview in an iterative process, allowing early insights to be explored more fully in later interviews and topic guides to be amended as necessary. A reflexive diary will be kept during the recruitment, interview and data analysis process to ensure transparency of the analysis process. Respondent validity and independent coding by two researchers will be conducted to check the validity of emergent themes.

### Proposed sample size

The sample size of 240 will provide adequate power to detect a standardised mean difference of 0.40 in the primary outcome (RMDQ; 5% significance, 80% power) assuming attrition of 20%. Using data from Critchley *et al* (2007) and our own initial feasibility study,^57^ ^30^ this equates to a 2-point difference between groups, where a 2–3 point difference in the RMDQ score is considered clinically important.^51^ It is hoped attrition will be minimised by a full explanation prior to recruitment of the time commitment required and importance of completing all follow-up questionnaires. A protocol will be developed to ensure an optimum and standardised follow-up process, including recording multiple contact addresses, email addresses and phone numbers.

### Statistical analysis

The statistical analysis plan has been approved by the Trial Steering Committee (TSC). The trial will determine the efficacy of the PACT intervention within six secondary care physiotherapy clinics. The main efficacy analysis will be performed only once the database has been cleaned and locked.

Stata 12.1 or higher will be used for the descriptive and main inferential analyses. The main efficacy analysis for primary, secondary and process outcomes will follow an intention-to-treat framework, whereby participants are analysed according to the groups to which they were randomised. The analysis will be conducted by the trial statistician (SN) blind to group allocation. SN will only be unblinded once the main efficacy analysis has been completed. Between-group differences (treatment efficacy) will be estimated for the primary outcome RMDQ at the postintervention 3-month and 12-month follow-up assessments using linear mixed-effects models. Random effects for the intercept and time will be included in the model. Treatment group, time and a treatment by time interaction term will be included as covariates to allow estimates of treatment effect at each time point to be calculated. In addition, a random effect for physiotherapist will be included to account for the partial clustering within physiotherapists in the intervention arm. Estimation of the treatment effects on the secondary and process outcomes will employ the same method as the primary efficacy analysis. All outcome variables are continuous.

Planned secondary analysis will be performed to determine whether the treatment effect occurs via changes in the process variables as hypothesised (pain acceptance and committed action). Specifically, the proportion of the treatment effect for disability (RMDQ), QoL (WSAS) and mood (PHQ-9 and GAD-7) at each follow-up that is mediated by the treatment effect on the process variables at 3 months will be determined. This will be estimated by the product of coefficients method using bootstrapped SEs.^57^ This analysis will be undertaken irrespective of achieving the statistical significance. Where the treatment effect is non-significant, additional further analysis will be conducted to determine the role of post-randomisation effect modifiers in the negative result (adherence, treatment fidelity and therapeutic alliance). For example, should there be considerable non-adherence to the treatment, the efficacy of the treatment for those who adhere to treatment will also be estimated in terms of the complier average causal effect (CACE).

## Ethics and dissemination

### Ethical issues

The trial will be conducted in accordance with current guidelines for ethical research conduct and subject to full Research Ethics Committee (REC) approval (National Research Ethics Committee South Central—Berkshire; 14/SC/0277), including any provisions of site-specific assessment, and local research and development approval. It will comply with the International Conference for Harmonisation of Good Clinical Practice (ICH GCP) guidelines and the Research Governance Framework for Health and Social Care. The trial is registered on a trial registry (ISRCTN95392287) and the lead site (Guy's & St Thomas' NHS Foundation Trust; GSTT) will audit this project annually to ensure compliance with the necessary legislation.

All patients in the trial will benefit from receiving physiotherapy for their CLBP. This is a very low-risk study as both treatments are non-invasive and delivered by appropriately qualified physiotherapists. Patients attending PACT sessions should visit hospital less often and will receive additional resources to aid self-management of their condition, possibly reducing its impact on participants' lives. This may lead to benefits for society and the NHS, such as reduced healthcare usage and less time off work, which is important in such a widespread and costly condition. The disadvantage of taking part is the additional time spent completing the questionnaires and for some patients an interview. These should not take more than 60 min in total to complete. Potential participants will be fully informed of the trial procedures before entering the study via a patient information sheet.

#### Informed consent

Potential participants will be identified by clinical physiotherapists from each hospital centre, informed about the RCT in writing and invited to participate. The physiotherapist will explain that participation is completely voluntary and that they are free to refuse involvement. They will be given at least 24 hours to consider whether they would like to participate. The RAs will then contact them to see if they are interested in participating and answer any questions about the study, prior to conducting the screening process and signing the consent form.

#### Fair access

Any adult patient referred to physiotherapy with low back pain lasting over 12 weeks and good English will be eligible for the trial. Participants will be able to complete measures online, by post or in person, so they should not be disadvantaged if they do not have access to the internet.

### Dissemination

The results of this study will be communicated to participants at the end of the study and disseminated via peer-reviewed publications, patient interest groups and conference presentations. The results will enable clinicians, patients and health service managers to make informed decisions regarding the efficacy of PACT for patients with CLBP. However, further studies will be necessary to demonstrate the generalisability of the findings beyond physiotherapy services in London and the South East, as well its effectiveness and cost-effectiveness.

### Service user involvement

CLBP patients have been involved in the design of the PACT trial and service users have contributed to the development of the patient guide. Participants from the feasibility study have also provided input and feedback on the proposals for this RCT. One of them is now the patient and public involvement (PPI) representative for this study, providing ongoing input (informal feedback and participating in TSC meetings) to ensure it addresses issues relevant to users.

### Research governance

This study will be conducted in accordance with the ICH GCP guidelines and the Research Governance Framework for Health and Social Care. King's College London is the sponsor of the RCT.

The TSC will meet every 6 months to oversee the trial procedures and ensure good conduct of the study. The TSC has an independent chair and two independent members plus a PPI representative. The trial management team (EG, DC, LM and the RAs) will hold monthly meetings to ensure the smooth running of the trial. The RAs will circulate a monthly newsletter to stakeholders to review progress relative to the project plan and highlight any issues that need to be addressed. Members of the team will consult each other immediately by email and/or phone about any issues that arise between meetings.

### Monitoring and auditing

The study will be monitored and audited in accordance with King's College London procedures. All trial-related documents will be made available on request for monitoring and audit by King's College London, trial NHS partners, the REC and other licencing bodies.

### Assessment of safety

All patients will be assessed and treated by an experienced grade 6 or 7 physiotherapist.

#### Adverse events

Any adverse events will be recorded by the treating physiotherapist in the clinical notes and reported to the RA and chief investigator immediately via email. Patients will also be offered the opportunity to report any adverse events on the follow-up questionnaires. If a patient becomes distressed during treatment, then the PACT physiotherapists will be adequately trained to deal with this or to identify a need for more input/support and refer them for an appropriate assessment. There will be clinical supervisors available at each research site if needed, and experienced psychologists will supervise physiotherapists delivering PACT on a monthly basis.

#### Serious adverse events

An adverse event is defined as serious if it results in an outcome which is life-changing/threatening, disabling or incapacitating. Any serious adverse events that are recorded will be immediately referred to the chief investigator, who will assess whether it is an adverse reaction that is classed as serious, whether it could have been caused by the intervention and whether it is unexpected. Any serious adverse reactions will be reported to the TSC for monitoring and advice. They will advise whether the participant should be withdrawn from either their randomised treatment or from the trial. Arrangements will be made by the trial team for further assessment and management as agreed with the relevant authorities, GP and participant. A report of the outcome will be provided to the TSC within 1 month.

#### Stopping rules

The trial may be stopped prematurely by the sponsor or chief investigator on the basis of new safety information or for other reasons given by the TSC, regulatory authority or ethics committee concerned. The trial may be halted on the advice of the TSC if recruitment rates are substantially below expected levels with no possibility of remedial action or if there are serious adverse reactions attributable to the trial which mean it is unsafe to continue. If the study is terminated prematurely, active participants will be informed and no further participant data will be collected.

### Data storage

Data will be collected and retained in accordance with the Data Protection Act 1998. The Data Protection Policy of King's College London will be complied with. The responses to questionnaires will be stored in an anonymised form on a password-protected university computer. The anonymised paper questionnaires will be stored in a locked filing cabinet at Guy's Campus, King's College London. Study documents (paper and electronic) will be retained in a secure location during and after the trial has finished. All source documents will be retained for a period of 5 years following the end of the study. The chief investigator will be the custodian of the data and the data will only be used by the study team.

### Conclusions

This paper describes the protocol for the PACT study. The PACT study RCT will assess the feasibility and acceptability of delivering a novel psychologically informed physiotherapy intervention with staff and patients. It is the first trial to test the efficacy of an ACT-informed, physiotherapist-delivered intervention for CLBP. It is noted that further studies will be necessary to demonstrate the generalisability of the findings beyond physiotherapy services in London and the South East, as well its effectiveness and cost-effectiveness.
